# Clinical Implications of Inflammation in Patients With Cirrhosis

**DOI:** 10.14309/ajg.0000000000003056

**Published:** 2024-08-27

**Authors:** Victoria T. Kronsten, Debbie L. Shawcross

**Affiliations:** 1 Institute of Liver Studies, Department of Inflammation Biology, School of Immunology and Microbial Sciences, Faculty of Life Sciences and Medicine, King's College London;; 2 Institute of Liver Studies, King's College Hospital, Denmark Hill, London.

**Keywords:** systemic inflammation, cirrhosis, liver disease

## Abstract

Cirrhosis-associated immune dysfunction refers to the concurrent systemic inflammation and immunoparesis evident across the disease spectrum of chronic liver disease, ranging from the low-grade inflammatory plasma milieu that accompanies compensated disease to the intense high-grade inflammatory state with coexistent severe immune paralysis that defines acute decompensation and acute-on-chronic liver failure. Systemic inflammation plays a crucial role in the disease course of cirrhosis and is a key driver for acute decompensation and the progression from compensated to decompensated cirrhosis. Severe systemic inflammation is fundamental to the development of organ dysfunction and failure and, in its most extreme form, acute-on-chronic liver failure. Systemic inflammation propagates the development of hepatic encephalopathy and hepatorenal syndrome-acute kidney injury. It may also be involved in the pathogenesis of further complications such as hepatocellular carcinoma and mental illness. Those patients with the most profound systemic inflammation have the worst prognosis. Systemic inflammation exerts its negative clinical effects through a number of mechanisms including nitric oxide-mediated increased splanchnic vasodilation, immunopathology, and metabolic reallocation.

## Graphical abstract

**Figure:**
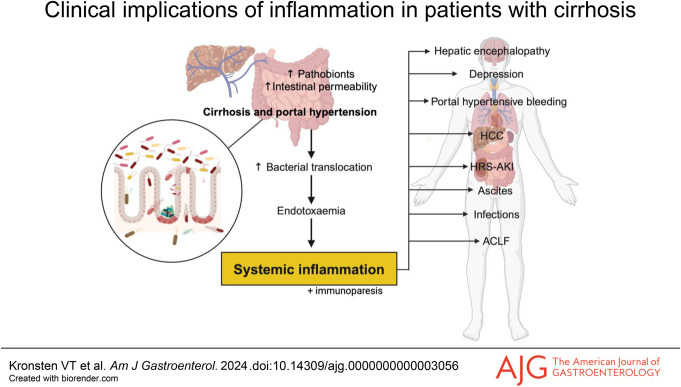

## INTRODUCTION

Cirrhosis-associated immune dysfunction (CAID) describes the continuum of immune alterations observed across both the innate and humoral compartments in cirrhosis comprising concurrent systemic inflammation and immunoparesis (1,2).

Attention on the role of systemic inflammation in cirrhosis grew after Rolando et al (2000) uncovered the importance of the systemic inflammatory response syndrome (SIRS) (3) in acute liver failure, irrespective of infection, and demonstrated that severity of SIRS was associated with multiorgan failure and death (4). This led to a vast number of studies exploring systemic inflammation as a potential mechanism for acute decompensation (AD) in cirrhosis and the development of acute-on-chronic liver failure (ACLF) (5–9). Systemic inflammation in cirrhosis is evidenced by increased levels of acute phase proteins (8), endothelial markers of activation (10), proinflammatory cytokines and their receptors (11,12), markers of macrophage activation (13), systemic oxidative stress, and heightened expression of surface activation antigens on circulating immune cells (13–16).

CAID is a dynamic process; it involves 2 different immune phenotypes and progresses in tandem with cirrhosis stage (16). A low-grade systemic inflammatory phenotype, systemic inflammation without immunoparesis, is present in patients with compensated cirrhosis and non-acute decompensated disease without organ failure (17). The high-grade inflammatory phenotype, exhibited in ACLF, encompasses severe systemic inflammation and profound immune paralysis, with effector cells unable to host a response again invading pathogens (16).

The development of systemic inflammation in cirrhosis is gut-derived, resulting from translocation of bacteria and pathogen associated molecular patterns (PAMPs), such as lipopolysaccharide, across a compromised gut barrier into the portal and systemic circulation, in the absence of overt bacterial infection (8,18). PAMPS are recognized by pattern recognition receptors (PRRs), expressed on gut-associated lymphoid tissue, mesenteric lymph nodes, and hepatic macrophages, which activate innate immune cells and induce the synthesis of proinflammatory cytokines (15,19). Reduced gut microbial diversity with an increase in pathobiont species coupled with the increased intestinal permeability that develops in cirrhosis further encourage this translocation, augmenting systemic inflammation (20). Metagenomic studies have demonstrated that human gut microbiota alterations are associated with complications in cirrhosis (21).

CAID predisposes patients with cirrhosis to develop bacterial and fungal infections and is a key mediator in cirrhosis progression, development of complications and prognosis (22). The degree of CAID correlates with bacterial translocation, liver disease severity, and organ failure (15).

## DISEASE STAGES IN CIRRHOSIS

**Figure 1.**
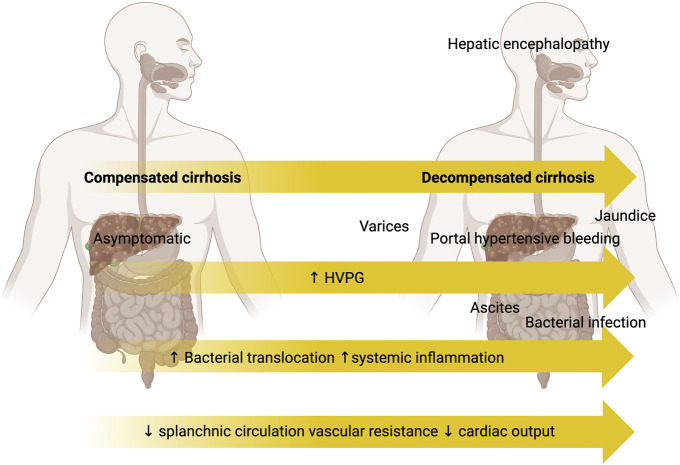
Disease stages in cirrhosis. The natural history of cirrhosis comprises 2 key phases—compensated cirrhosis, a long asymptomatic phase, followed by decompensated cirrhosis. Decompensated cirrhosis is characterized by complications of synthetic dysfunction and portal hypertension; ascites development, portal hypertensive bleeding, hepatic encephalopathy, jaundice, and infection. This is driven by rising portal pressure (measured by HVPG) and increased bacterial translocation and resultant systemic inflammation, accompanied by decreased splanchnic circulation vascular resistance and decreased cardiac output. *Created with* biorender.com. HVPG; hepatic venous pressure gradient.

The natural history of cirrhosis comprises 2 phases (Figure 1). Compensated cirrhosis describes a usually long asymptomatic phase, followed by decompensated cirrhosis which is a rapidly progressive disease process characterized by complications of portal hypertension and synthetic dysfunction (ascites, variceal bleeding, hepatic encephalopathy, and jaundice) (23).

Decompensation and development of gastroesophageal varices do not usually occur until the portal pressure has reached the threshold of significant portal hypertension (defined by hepatic venous pressure gradient ≥10 mm Hg) (24). Progression of liver disease with increasing portal pressure is associated with bacterial translocation and systemic inflammation (15), alongside neurohormonal alterations resulting from decreased splanchnic circulation vascular resistance and decreased cardiac output (25).

Decompensation presents as AD in a proportion of patients (26). AD refers to the sudden development of one or more major complications of decompensated cirrhosis and usually necessitates inpatient management (27). A further definition of non-AD (NAD) (26) has recently been introduced, referencing that the first decompensating event for almost half of patients with cirrhosis is non-acute, with slow development of ascites or grade 1 or 2 hepatic encephalopathy, and does not require hospitalization, though is still associated with increased mortality (17). Decompensated cirrhosis is defined by multiple episodes of AD, often post initial NAD (17,26), during which patients are particularly susceptible to infection, such that some definitions include bacterial infection as part of the AD process (5). ACLF is a distinct syndrome that occurs in acutely decompensated patients and is characterized by high-grade systemic inflammation, one or more organ failures and a high 28-day mortality (28).

## ACUTE DECOMPENSATION AND PROGRESSION TO ACLF

Episodes of AD, be it ascites, hepatic encephalopathy, or portal hypertensive bleeding, were previously believed to develop through distinct pathophysiological mechanisms. However, systemic inflammation has recently been proposed as a key unifying driver of all acute decompensating events, and thus, the transition from compensated to decompensated cirrhosis, either acting alone or in synergy with organ-specific mechanisms (5), such as hyperammonemia in hepatic encephalopathy (29). Acute spikes in systemic inflammation before AD may, for example, result in a final rise in portal pressure to trigger variceal hemorrhage (5), and serum interleukin-6 (IL-6) concentration has been demonstrated as an independent predictor of decompensation in outpatients with cirrhosis (30).

Trebicka et al (2019) demonstrated that, while all patients with AD have some degree of systemic inflammation, patients who go on to develop ACLF have more sustained systemic inflammation, demonstrated by raised IL-6, IL-8, IL-1 receptor antagonist, and human non-mercaptalbumin 2 (31).

Prior AD is a risk factor for future ACLF development, and this may be linked to inflammasome activation demonstrated by higher baseline levels of ILs (32).

The PREDICTing Acute-on-Chronic Liver Failure (PREDICT) study was a European prospective observational study of 1,071 patients with AD of cirrhosis, defined as the acute development of ascites, gastrointestinal hemorrhage, hepatic encephalopathy, and/or infection requiring hospitalization, with the aim of characterizing the course of AD and identifying predictors of ACLF (33). Three prognostic subgroups were identified—stable decompensated cirrhosis (SDC, no death during hospitalization and no rehospitalization during early 3-month follow-up), unstable decompensated cirrhosis (UDC, death by any cause apart from ACLF during first hospitalization or minimum of one admission during early 3-month follow-up), and pre-ACLF (ACLF development during 3-month follow-up). Three-month and 1-year mortality rates increased in line with AD group severity, with pre-ACLF having the highest mortality rates of 53.7% and 67.4%, respectively. The 3 clinical courses were unrelated to cirrhosis etiology, or continued alcohol consumption in patients with alcohol-related cirrhosis, suggesting a different unifying mechanism. Of note, the 3 groups differed significantly with respect to degree and course of systemic inflammation; pre-ACLF patients exhibited high-grade systemic inflammation at enrolment, with increased levels of white cell count and C-reactive protein (CRP), which increased dramatically during follow-up coinciding with progression to ACLF, while UDC patients displayed low-grade inflammation at enrolment which remained stable during follow-up showing no clear increase or decrease. Patients with SDC also exhibited low-grade inflammation akin to the UDC group at enrolment, but this decreased rapidly during follow-up (32). Thus, patients with SDC developed an index episode of AD in the context of moderate systemic inflammation, which decreased, and all patients recovered from this episode. Patients with pre-ACLF, however, acutely decompensated in the setting of greater systemic inflammation, which increased further with ACLF development. The pre-ACLF group presented with a more severe disease state at enrolment, with a higher prevalence of renal dysfunction, encephalopathy, ascites, and bacterial infections, and had an accelerated course from development of decompensated cirrhosis to transplantation or death than those in the SDC group (12 vs 20 months, respectively). Patients with UDC did not exhibit such severe systemic inflammation at enrolment or a clear increase during follow-up, likely explaining why they did not develop ACLF, but had higher prevalence of features indicative of severe portal hypertension suggesting this as a second pathophysiological mechanism of AD (33). Thus, the PREDICT study confirmed that acutely decompensated patients with the greatest systemic inflammation are most at risk of ACLF development and death.

NAD describes the slow and progressive development of complications of cirrhosis in the outpatient setting (26). NAD comprises a separate distinct pathway to the development of decompensated cirrhosis compared with AD and is likely driven more by portal hypertension than systemic inflammation (17); however, further work is required in this area.

## ASCITES AND GASTROINTESTINAL BLEEDING

In the PREDICT study, the development of ascites, the decompensating event associated with the most extensive organ dysfunction (34), was associated with a higher-grade of systemic inflammation compared with hepatic encephalopathy and gastrointestinal hemorrhage (33).

Serum bacterial DNA levels and the severity of systemic inflammation correlate with the degree of portal hypertension in cirrhosis (35). A study of patients with spontaneous bacterial peritonitis revealed that those with higher plasma levels of tumor necrosis factor (TNF)-α had increased portal pressure (34). Systemic inflammation activates toll-like receptors (TLRs) on hepatic stellate cells rendering them responsive to the increased levels of vasoconstrictors (5). Stellate cells alter intrahepatic vascular resistance (36). Kupffer cells, liver macrophages, are also activated in systemic inflammation through the TLR-4 complex on their cell surface (37) and release proinflammatory cytokines and reactive oxygen species (38). Oxidative stress reduces nitric oxide (NO) bioavailability resulting in further reactive oxygen species and peroxynitrite formation, alongside endothelial NO synthase inhibition (39). Thus, this imbalance in hepatic vasoconstrictor and vasodilator mechanisms in systemic inflammation may result in increased vascular resistance (40). The administration of high density lipoprotein, which is anti-inflammatory and neutralizes circulating lipopolysaccharide, restored hepatic endothelial NO synthase activity and reduced portal pressure in a rat model of cirrhosis (41), lending further weight to this hypothesis (5).

A recent study by Zanetto et al (2023) of 169 patients with AD, demonstrated that inflammation severity, measured by CRP, is also predictive of nonportal hypertensive bleeding (42).

## HEPATIC ENCEPHALOPATHY

It is now widely accepted that systemic inflammation, frequently accompanied by infection, acts in synergy with hyperammonemia in the pathogenesis of hepatic encephalopathy (43,44). Systemic inflammation exacerbates hepatic encephalopathy in animal models (45–47) and patients with cirrhosis (44,48–50), with the degree of neurocognitive impairment correlating with inflammation levels, and patients with acute liver failure with greater levels of inflammation exhibit a more rapid progression in encephalopathy severity (4). Furthermore, studies have shown that the SIRS response, and not ammonia level, liver biochemistry nor liver disease severity, correlates with severity of hepatic encephalopathy and also survival (50).

In brief, circulating proinflammatory cytokines activate cerebral endothelial cells. Circulating immune cells can then adhere to the activated endothelium, be recruited by the brain parenchyma, and result in the activation of microglia and astrocytes (16,51,52). These resident immune cells then produce inflammatory mediators, such as proinflammatory cytokines, propagating neuroinflammation and altering neurotransmission and behavior (53). Systemic inflammation also disrupts tight junction protein regulation within the blood-brain barrier, increasing permeability. Activated microglia and astrocytes further contribute to blood-brain barrier dysfunction, further propagating this process resulting in clinical features of hepatic encephalopathy (54,55).

Increasing evidence also suggests that systemic inflammation resulting in central, or neuro, inflammation may, in part, explain the higher prevalence of depression in cirrhotic patients (18%–58%), compared with the general population (10%) (56–60). A cohort of patients with depression exhibit systemic immune activation, with increased levels of plasma proinflammatory cytokines, chemokines, and acute phase reactants, similar to that observed in cirrhosis (61,62). Increased levels of proinflammatory cytokines in the cerebrospinal fluid of depressed patients, and microglial activation in the brains of depressed patients postmortem, have been demonstrated (63,64). Animal models have identified 3 main cytokines (TNF-α, IL-1β, and IL-6) that facilitate peripheral to central communication in systemic inflammation through 4 pathways—neural mechanisms, cerebral endothelial cells, circumventricular organs, and peripheral immune cell-to-brain signaling (58,64). Such signals can then affect all central nervous system fields involved in the pathogenesis of depression and can act directly on the central nervous system cells implicated in depression; astrocytes and microglia (60).

## ACUTE KIDNEY INJURY AND HEPATORENAL SYNDROME

Acute kidney injury is one of the most common complications of cirrhosis. Systemic inflammation is central to its development, and bacterial infection is a well-known precipitant (65).

Hepatorenal syndrome-acute kidney injury (HRS-AKI, formerly Type 1 HRS) is driven by peripheral arterial vasodilation and cardiac dysfunction (25), which leads to reduced effective arterial volume and activation of the renin-angiotensin-aldosterone system and sympathetic nervous system. This results in afferent arteriolar constriction, further decline in renal blood flow and the development of HRS-AKI (66).

Systemic inflammation stimulates NO production in splanchnic arterioles. This worsens circulatory dysfunction, decreasing effective arterial blood volume further leading to increased vasoconstriction exacerbating renal hypoperfusion and dysfunction (15,25,66).

Renal failure is the most frequent organ failure in ACLF and is driven by high-grade inflammation (67), resulting in renal inflammation. Trawalé et al (2010) studied renal biopsies from patients with cirrhosis and found that fibrosis and interstitial inflammation (from mononuclear and polymorphonuclear leucocytes) were the most common abnormalities, with inflammation associated with renal failure (68). A further study revealed tubular injury and apoptosis with overexpression of TLR4 on epithelial tubular cells and increased urinary excretion of TLR4 in renal biopsies from patients with cirrhosis and renal dysfunction (69).

Thus, the mechanisms underpinning the development of acute kidney injury in cirrhosis are similar to those that lead to acute kidney injury in sepsis. Circulating PAMPs, damage-associated molecular patterns (DAMPs), and cytokines damage the glomerular endothelial glycocalyx aiding the transmigration of activated leucocytes into the peritubular interstitium (5). Inflammation extends to the epithelial tubular cells which experience hypometabolism because nutrients are reallocated to energy consuming activated immune cells. This results in reduced tubular function, greater sodium release, glomerulus-tubular feedback mechanism activation, angiotensin II secretion, afferent arteriolar vasoconstriction, and reduced glomerular filtration rate (5).

The contribution of varying degrees of systemic inflammation to the development of acute kidney injury in ACLF is supported by Piano et al (2018) who found the renal response to terlipressin and albumin is dependent on ACLF grade, with 60% resolution of HRS in patients with ACLF-1, but only 29% resolution in those with ACLF-3 (67). It is likely that renal vasoconstriction secondary to effective arterial hypovolemia and renal inflammation occur concurrently in HRS and thus in patients with low-grade systemic and renal inflammation, such as ACLF-1, HRS responds to hemodynamic optimization with terlipressin and volume expansion. However, in patients with ACLF-3 HRS is presumed to relate more to renal inflammation, and therefore, standard treatment is less effective (5).

## CARDIAC DYSFUNCTION

Systemic inflammation is also related to cardiac dysfunction in cirrhosis. Reduced heart rate variability correlates with severity of decompensation while inversely correlating with CRP and white cell count (70). Further studies have demonstrated that cardiopulmonary hemodynamics and CRP are predictive of decompensation, and death or need for transplant in those already decompensated (71), and that cardiac index and IL-6 levels are predictive of fatal ACLF development (72).

## ACUTE-ON-CHRONIC LIVER FAILURE

ACLF is a severe complex syndrome characterized by AD in cirrhosis, organ failure(s), and a high short-term mortality at 28 days approaching 30%. The landmark European Association for the Study of the Liver-Chronic Liver Failure Consortium Acute-on-Chronic Liver Failure in Cirrhosis study, that involved 1,343 European patients hospitalized for AD, provided the first evidence-based definition of ACLF and demonstrated that patients with ACLF have more severe bacterial infections and greater systemic inflammation, demonstrated by raised leucocytes and CRP (6). Furthermore, leukocyte count was an independent predictor of mortality in ACLF. ACLF is classified into 3 severity grades (ACLF-1, 2, and 3), depending on the number of organ failures. The intensity of the inflammatory response further increased with severity of ACLF grade (6). High-grade systemic inflammation is the key driver of ACLF, with greater levels of proinflammatory cytokines, chemokines, adhesions molecules, and leucocyte migration, with severity of inflammation correlating with prognosis (31,33,42,73,74).

**Figure 2.**
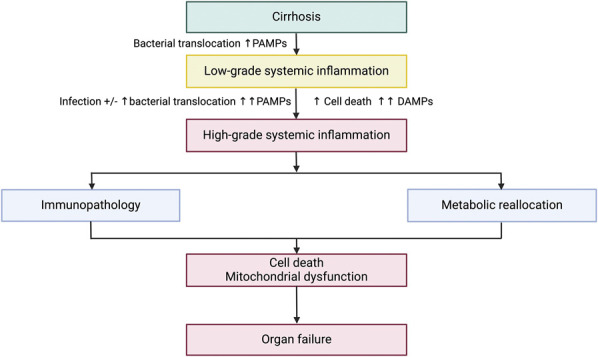
Role of systemic inflammation in the development of organ failure in cirrhosis. Increased bacterial translocation results in increased PAMPs which trigger low-grade systemic inflammation in cirrhosis. A precipitating event, such as bacterial infection, spikes of bacterial translocation and/or increased hepatocyte death lead to a further rise in PAMPs and DAMPs causing high-grade systemic inflammation. Both immunopathology, direct immune-mediated tissue damage, and metabolic reallocation, counteraction of the nutrient deficit caused by the activated immune system, lead to cell death and mitochondrial dysfunction resulting in organ failure. *Created with* biorender.com. DAMPS, damage-associated molecular patterns; PAMPS, pathogen-associated molecular patterns.

The excessive inflammatory response in ACLF induces direct tissue damage, a process termed immunopathology (75) and leads to organ failure (Figure 2). Proinflammatory cytokines damage the endothelium glycocalyx and stimulate neutrophil and monocyte adhesion to endothelial cells and their subsequent migration into tissues (76). These activated immune tissues then emit mediators, such as leukotrienes, prostaglandins, proteases, and cytotoxic cytokines causing cell death and further direct tissue injury (68). Patients with ACLF may also exhibit a decrease in the capacity of peripheral organs to endure such an inflammatory response, and this may be more marked in patients who have not previously experienced decompensation, furthering immune-mediated tissue damage (6,75).

Recent work has revealed that metabolic reallocation also occurs in systemic inflammation. Activated innate immune cells have a high metabolic demand, as demonstrated through blood metabolomic work in sepsis and ACLF, and circulating nutrients (fatty acids, amino acids, glucose) are redirected to such cells from peripheral organs (77–80). This results in reduced mitochondrial oxygen consumption and adenosine triphosphate production in peripheral organs leading to dysfunction and failure (Figure 2) (5).

The liver is a component of the innate immune system. PAMPs and proinflammatory cytokines induce increased hepatic synthesis of acute-phase proteins (81). Thus, the liver is also a site of vast energy-consuming anabolic metabolism in systemic inflammation. A reduction in negative acute-phase protein synthesis, such as transferrin and albumin (81), alongside the inhibition of hepatic biotransformation (enzymatic transformation of lipophilic molecules, such as bilirubin, into water soluble molecules) occurs in systemic inflammation to negate the increased energy used in positive acute-phase protein synthesis (82). In sepsis, proinflammatory cytokines inhibit hepatic biotransformation which results in hyperbilirubinemia and jaundice (82). Together, this suggests that metabolic reallocation also occurs within the liver itself, resulting in deterioration in liver function (5).

The trigger leading to immune activation and immunopathology remains to be determined; however, 33% of ACLF cases occur in the presence of bacterial infection (83). Infecting bacteria release PAMPs, unique molecular structures that are recognized by host PRRs, such as TLRs, on the cell surface and NOD-like receptors present in the cytosol of the cell (84). PRRs then activate transcription factors, such as nuclear factor-kappa B and activator protein 1 (85), which sequentially stimulate the expression of genes encoding proinflammatory molecules, such as TNF-α and IL-6 (86). PAMPs can also translocate from the intestinal lumen to the systemic circulation in the absence of overt bacterial infection, with resultant activation of the aforementioned inflammatory pathway. Bacterial overgrowth, impaired intestinal immune system function, and increased intestinal permeability have all been demonstrated in patients with AD who develop ACLF, substantiating this hypothesis (87,88).

In the absence of bacterial infection and/or bacterial translocation DAMPs, released by injured, dying, or dead cells may stimulate the systemic inflammation seen in ACLF by binding to specific PRRs (84). Necrosis and other inflammatory types of cell death, such as pyroptosis and necroptosis, are more common in cirrhosis increasing the release of DAMPs (89). Host genetic factors, such as single-nucleotide variants, may play a role in the inflammatory response of the host immune system to PAMPs and DAMPS (90).

## INFECTION

**Figure 3.**
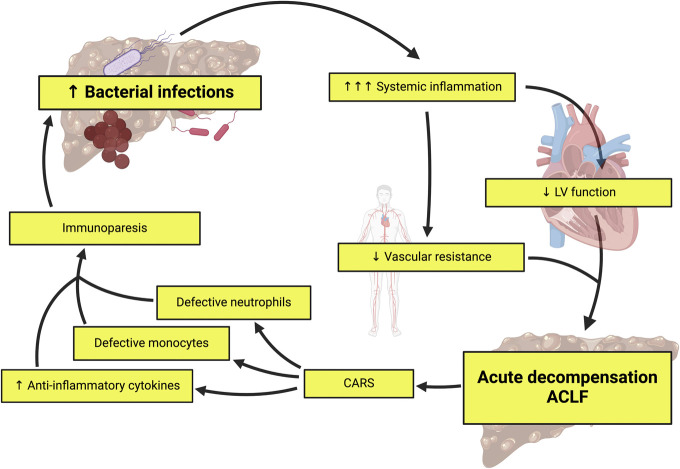
Bacterial infections are common in cirrhosis and precipitate complications. Bacterial infections in cirrhosis exacerbate existing systemic inflammation which impairs left ventricular function and reduces splanchnic and systemic circulation vascular resistance. This leads to acute decompensation and ACLF. The CARS develops in acute decompensation and ACLF to minimize the effects of the intense proinflammatory response. The CARS induces immunoparesis through a number of mechanisms; levels of circulating anti-inflammatory cytokines increase, monocytes exhibit features of defective anti-bacterial function and a range of neutrophil functions are impaired. The resultant immunoparesis thus impairs the host response to pathogens and predisposes patients with cirrhosis to infection. *Created with* biorender.com. ACLF, acute-on-chronic liver failure; CARS, compensatory anti-inflammatory response syndrome; LV, left ventricle.

Bacterial infections are common in cirrhosis and frequently precipitate AD and/or the development of ACLF. In the PREDICT study, 1 in every 3–4 patients per group had an infection at the time of decompensation (33), and further work revealed proven bacterial infections, alongside severe alcohol-related hepatitis, were the most common precipitants to both AD and ACLF (91). Two mechanisms have been described to explain this association (Figure 3).

First, the augmented systemic inflammation caused by bacterial infections in cirrhosis, as seen in sepsis, may precipitate decompensation, impair left ventricular contractibility, and reduce splanchnic and systemic circulation vascular resistance (15,40). Thus, the exaggerated inflammatory response to bacterial infections in cirrhosis frequently precipitates AD, HRS-AKI, ACLF, and death (92,93). Furthermore, peritoneal immunity is also related to systemic inflammation. Type-1 interferon responses, triggered by PAMPs, prime peritoneal macrophages, and regulate caspase-5-mediated progranulin release during spontaneous bacterial peritonitis, and higher levels of progranulin are associated with lower 90-day transplant-free survival after spontaneous bacterial peritonitis (94).

Second, it may be that the increase in bacterial infections is a consequence of the compensatory anti-inflammatory response syndrome, which leads to immunoparesis, the second element of CAID (95,96). Immunoparesis describes the impaired antibacterial function of immune cells and is seen in the most severe end of the CAID spectrum, notably in patients with ACLF. 65% of patients with AD-ACLF enrolled in the Consortium Acute-on-Chronic Liver Failure in Cirrhosis study developed an infection during the 28-day follow-up period (6). The mechanisms through which immunoparesis develops are many. Circulating levels of anti-inflammatory cytokines that induce immunoparesis, such as IL-10 (IL-10) and IL-1 receptor antagonist, are markedly raised in patients with AD and more so in those with ACLF (74). Circulating monocytes also exhibit features of defective antibacterial function in AD, and such cells are more prevalent in those with ACLF (97,98). In decompensated cirrhosis, neutrophil migration, recognition of bacteria and subsequent phagocytosis, degranulation, respiratory burst, and generation of neutrophil extracellular traps is impaired (99). Thus, immunoparesis occurs as a compensatory mechanism in AD, to minimize the effects of the intense proinflammatory response. Consequently, immunoparesis increases the frequency of bacterial infections (5,100).

## HEPATOCELLULAR CARCINOMA

Chronic inflammation in cirrhosis may also be involved in the pathogenesis of hepatocellular carcinoma (HCC) (101). Increased IL-6 which leads to overactivation of signal transducer and activator of transcription 3, a transcriptional activator that has been shown to contribute to the tumorigenesis of HCC, has been demonstrated in patients with HCC and preclinical models (102,103). TNF-α which leads to nuclear factor-kappa B and Jun N-terminal kinase activation has also been shown to promote HCC development in mouse models through inflammation, hepatocyte death, and subsequent proliferation (104).

## PROGNOSIS

While there is clear evidence for the role of systemic inflammation in the development of AD and ACLF, and its detrimental effect on prognosis, a recent study has also demonstrated that inflammatory markers correlated with mortality in 149 patients with a new diagnosis of cirrhosis. 20 markers, including leukemia inhibitory factor, a member of the IL-6 family, IL-6, and IL-8, correlated with 180-day mortality in this patient cohort, although there was no correlation with liver-related admissions (105).

## THERAPEUTIC INTERVENTIONS

Treatment strategies to reduce the systemic inflammation observed in cirrhosis, to treat AD and, ideally, prevent decompensation and the development of complications from the outset, are now critical. Established and developmental therapies are summarized in Table 1.

**Table 1.**
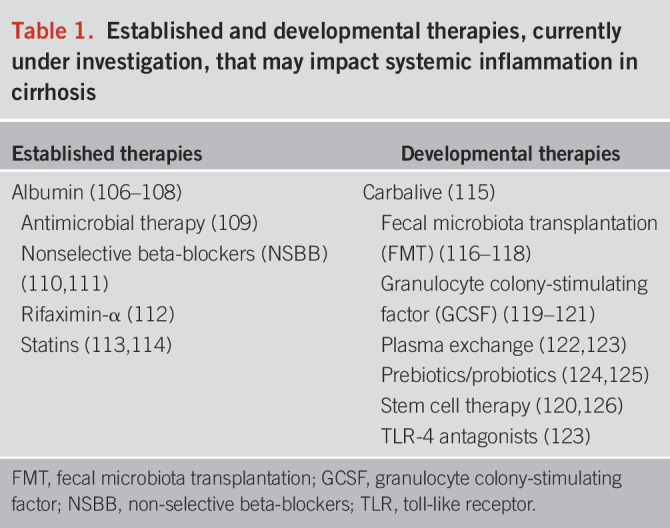
Established and developmental therapies, currently under investigation, that may impact systemic inflammation in cirrhosis

## CONCLUSION

**Figure 4.**
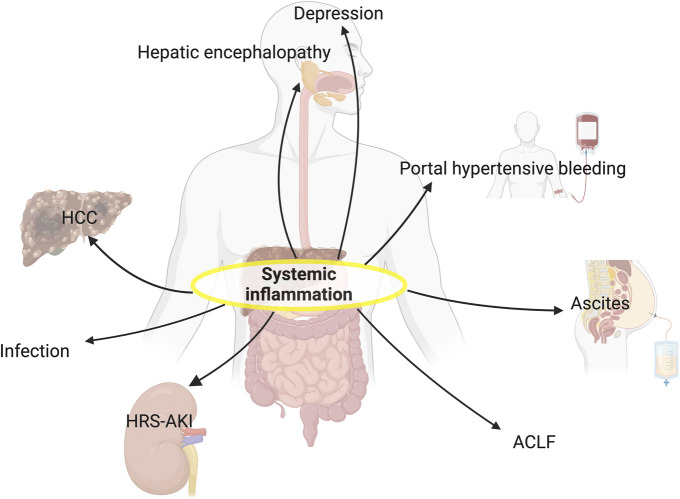
Clinical implications of systemic inflammation in cirrhosis. Systemic inflammation is a distinct feature of cirrhosis and acts in synergy with other pathophysiological mechanisms leading to acute decompensation; hepatic encephalopathy, portal hypertensive bleeding, and ascites. It is also central to the development of HRS-AKI and infection, notably when accompanied by immunoparesis. High-grade systemic inflammation, at the most severe end of the spectrum, triggers the development of ACLF. Systemic inflammation may also play a role in HCC development and depression. *Created with* biorender.com. ACLF, acute-on-chronic liver failure; HCC, hepatocellular carcinoma; HRS-AKI, hepatorenal syndrome–acute kidney injury.

Systemic inflammation plays a pivotal role in the clinical course of cirrhosis (Figure 4) and is a key driver of progression from compensated to decompensated cirrhosis and repeated AD. Severe systemic inflammation is intrinsic to the development of single and multiple organ failures and, in its most extreme form, ACLF. Systemic inflammation is also fundamental to the development of further complications in cirrhosis, such as HRS-AKI, and may be involved in the pathogenesis of HCC development and mental illness. The severity of systemic inflammation is closely related to prognosis; inflammatory markers correlate with mortality even in compensated disease. Therapeutic interventions that reduce inflammation without inducing immunosuppression are paramount and remain one of the main therapeutic challenges in cirrhosis.

## CONFLICTS OF INTEREST

**Guarantor of the article:** Victoria T. Kronsten, MBBS, BSc, MRCP.

**Specific author contributions:** V.T.K.: drafted the manuscript. D.L.S.: revised the manuscript. Both authors reviewed and approved the final draft submitted.

**Financial support:** None to report.

**Potential competing interests:** V.T.K. has no conflicts of interest to disclose. D.L.S. is the Principal Investigator of an Investigator-initiated study funded to King's College London by Norgine. D.L.S. has also undertaken consultancy for Norgine, EnteroBiotix, Satellite Bio, MRM Health and Apollo Therapeutics.
